# A Decomposition Analysis of Inequality in Malnutrition among Under-Five Children in Iran: Findings from Multiple Indicator Demographic and Health Survey, 2010

**Published:** 2019-04

**Authors:** Abdollah ALMASIAN KIA, Sahar GOODARZI, Heshmatollah ASADI, Ardeshir KHOSRAVI, Aziz REZAPOUR

**Affiliations:** 1. Health Management and Economics Research Center, Iran University of Medical Sciences, Tehran, Iran; 2. Department of Health Economics, School of Management and Information Sciences, Shiraz University of Medical Sciences, Shiraz, Iran; 3. Department of Public Health, School of Health and Nutrition, Lorestan University of Medical Sciences, Khorramabad, Iran; 4. Deputy of Public Health, Ministry of Health and Medical Education, Tehran, Iran

**Keywords:** Malnutrition, Inequality, Socioeconomic factors, Decomposition, Iran

## Abstract

**Background:**

Nutritional status at the early stages of children’s lives is essential for growth and development not only in infancy but also in adult life. This study aimed to measure the inequality in malnutrition among under-five children in Iran and explore the impact of socioeconomic factors on this inequality using a regression-based decomposition approach.

**Methods:**

Data were extracted from Iran’s Multiple-Indicator Demographic and Health Survey 2010. The concentration index of stunting, underweight, and wasting were applied in order to measure the magnitude of socioeconomic inequality in child malnutrition. Moreover, the concentration indices were decomposed to understand the contribution of socioeconomic variables in childhood malnutrition inequality.

**Results:**

The obtained concentration indices of stunting, underweight, and wasting were respectively −0.177, −0.092, and −0.031. Socioeconomic inequality in stunting and underweight was statistically significant, however this socioeconomic gradient was not observed in wasting. More than 50% of the inequality in stunting and about 63% of the inequality in underweight were influenced by socioeconomic status. Furthermore, maternal education was associated with 19% and 22% of inequality in stunting and underweight respectively.

**Conclusion:**

The average reduction of malnutrition indices at the national level hides the burden of malnutrition among children in poor families. If government and policymakers seek to solve this problem, they have to take direct and targeted actions to eliminate the existing inequalities in the socioeconomic determinants associated with malnutrition.

## Introduction

Despite the significant improvements in children’s health and the reduction of the overall rate of malnutrition in the world, developing countries are still bearing a great burden of malnutrition and it has remained as a challenge to public health in these countries (1). Globally, 156 million under-five years old were estimated to be stunted and approximately 50 million children under-five years of age were wasted. This rate of malnutrition is not equally distributed among countries; about 145 million stunted as well as 48 million wasted children are living in Asian and African countries (2). In addition to this inequality among countries, there are also socioeconomic inequalities within countries and children in lower social groups bear a greater burden of malnutrition.

Nutritional status of children is affected by many socioeconomic factors such as mother’s education and nutritional status, residential area, household wealth, and demographic characteristics (3–9). For the first time in Iran, the concentration index and decomposition method was used to analyze the socio-economic inequalities in child mortality at the national level and with regard to the provinces (10). Later, other researchers in Iran used this method to analyze socioeconomic inequalities in mental health (11), the extent of using health care (12), and in mortality of children under five years of age (13). However, no study has ever been carried out to assess the socio-economic inequality in malnutrition of under-five-year-old children in Iran and to determine the factors affecting this inequality.

Since providing evidence on the factors affecting malnutrition and its distribution among socioeconomic groups can help policymakers to take actions for targeted and optimized allocation of resources and to carry out nutritional interventions, therefore, this study was the first attempt to identify and analyze the factors affecting socioeconomic inequalities in malnutrition of children under five in Iran.

## Materials and Methods

### Data

The data for this study were extracted from Iran Multiple-Indicator Demographic and Health Survey (IrMIDHS)-2010. In this survey, demographic, health, and socioeconomic information of 30960 households (21870 urban and 9090 rural households) were collected (14). Overall, 8443 children whose data on height, weight, age, and sex as well as the data related to their mothers’ characteristics and the socio-economic status of their families had been collected accurately and completely were entered the study in order to assess the socioeconomic inequalities in child malnutrition and to measure the factors affecting it.

### Measuring malnutrition

The anthropometric indicators such as height-for-age, weight-for-age, and weight-for-height in z-score form are widely used to measure the health of children, particularly for malnutrition. We used the WHO’s (15) growth chart (16) to measure height-for-age, weight-for-age, and weight-for-height z-scores. The children whose z-score value for height-for-age, weight-for age, and weight-for-height was less than -2 were respectively considered stunted, underweight, and wasted (15).

### Measurement of socioeconomic status

The wealth index was used as a proxy of household income in order to assess the socioeconomic status of the households. First, data on household assets including TVs, refrigerators, freezers, radios, cell phones, wristwatches, computers, laptops, microwaves, washing machines, vacuum cleaners, dish washers, and cars as well as home ownership and household characteristics including heating and cooling systems, type of fuel in the kitchen, access to the internet, a source of drinking water, having a bathroom, number of rooms, and toilets were arranged and classified. Then, using the statistical method of principal component analysis, the wealth index score was calculated for each household. Finally, to determine the socioeconomic rank of each household, the wealth index scores were divided into 5 quintiles (poorest, poor, middle, rich, and richest). The method for estimating wealth asset index has been described in details elsewhere (17).

### Inequality analysis

Concentration index was applied in order to measure the degree of socioeconomic inequality in child stunting, underweight, and wasting (CI). Kakwani et al (18) suggested the CI formula as follows:

(I) $$c=\frac{2}{n.\mu }\left[\sum_{i=1}^{n}y_{i}R_{i}\right]-1$$

where is the sample size, *μ* is the mean of stunting, underweight or wasting indices, *y*_i_ is the value of each of the indices of stunting, underweight or wasting in the *i^th^* person, and *R_i_* shows the rank of socio-economic status of the *i*^th^ person. In this study, Kakwani formula was used to estimate the concentration index of stunting, underweight, and wasting.

### Decomposition of concentration index

The concentration index was decomposed in order to understand the contribution of socioeconomic variables to inequality in childhood malnutrition. This decomposition of the concentration index works only if the regression model is linear (19, 20). Therefore, we used the continuous form of stunting and underweight as dependent variables and multiple linear regression was run to estimate the coefficient between socioeconomic determinants and child malnutrition.

For a continuous outcome variable, a linear regression model linking the outcome variable (y) to the set of k determinants can be represented as follows:

(II) $$y_{i}=\alpha +\sum_{k}\beta_{k}x_{k}+\varepsilon$$

In which *β_k_* represents the coefficient of each independent variable and *ɛ* is the error term.

If (*y*_i_) in equation (I) is replaced by its equal amount in equation (II), the concentration index for (*y*_i_) will be

(III) $$c=\sum_{k}\left(\frac{\beta_{k}{\bar{x}}_{k}}{\mu }\right)c_{k}+\frac{{\mathrm{CG}}_{\varepsilon }}{\mu }=C_{\hat{y}}+\frac{{\mathrm{CG}}_{\varepsilon }}{\mu }$$

Where ${\bar{x}}_{k}$ is mean of *x_k_*, *c_k_* is a concentration index for *x_k_*, and *GCɛ* is the generalized CI for the error term (*ɛ*).

In equation (3), the first part (deterministic component) includes elasticity $\left(\frac{\beta_{k}{\bar{x}}_{k}}{\mu }\right)$ which reflects the impact of explanatory variables on malnutrition, and *C_k_* which indicates the inequality of the distribution of determinant variables among socio-economic groups. The second part (non-deterministic component) refers to the inequality in malnutrition among socio-economic groups which cannot be explained by contributors ($\left(\frac{CG_{\varepsilon }}{\mu }\right)$).

Socioeconomic characteristics including sex, age (in months), and birth-order of children, number of under-five children, mother’s education, mother’s age at birth (years), residential area, household size, and socioeconomic status were entered in the multivariate linear regression model as independent variables. The effectiveness of independent variables on socio-economic inequality in malnutrition had been proven in previous studies (21–23).

All statistical analyses were conducted in Stata 14.1 (Stata Corp, USA).

## Results

### Descriptive statistics

Table 1 shows the descriptive statistics of the outcome and explanatory variables in the present study. On average, the values of height-for-age, weight-for-age, and weight-for-height z-scores were respectively 0.61, 0.38, and 0.04. Boys were a little more than the girls (51.10% vs. 48.9%). A significant percentage of the children (63.34%) were living in urban areas while 36.66% were living in rural areas. About 10% of the mothers were not formally educated. In addition, the average number of people in each family was 4.4 and the distribution of the children under study was almost the same in economic quintiles.

**Table 1:** Descriptive statistics

***Variable***	***Mean (SD)***	***Number (%)***	***Min***	***Max***
Child health outcome				
Height - for- age z-score	0.61 (1.13)		−3.0	4.9
Weight- for- age z-score	0.38 (1.05)		−4.1	4.9
Weight - for - height z-score	0.04 (1.11)		−4.9	4.0
Sex of child				
Male (reference)		4,314(51.1)	0	1
Female		4,129(48.9)	0	1
Child’s age (months)	30.4 (17.3)		0.3	60
Birth-order of child	2.2 (1.5)		0	15
Number of under 5 children				
< 2 (reference)		6,392(75.9)	0	1
≥ 2		2,027(24.1)	0	1
Mother’s education				
Illiterate (reference)		884(10.47)	0	1
Primary or secondary		4,215(49.9)	0	1
Higher		921(10.9)	0	1
Mother’s age at birth (years)				
< 20 (reference)		671(8)	0	1
20 – 29		4,999(59.2)	0	1
30 – 39		2,526(29.9)	0	1
≥ 40		247(2.9)	0	1
Area of residence				
Urban (reference)		5,348(63.3)	0	1
Rural		3,095(36.7)	0	1
Wealth quintile				
Poorest (reference)		1,689(20.0)	0	1
Poor		1,700(20.1)	0	1
Middle		1,694(20.1)	0	1
Rich		1,690(20.0)	0	1
Richest		1,670(19.8)	0	1
Household size	4.4(1.6)		2	17

### Frequency and inequality in child malnutrition

Table 2 shows the frequency and concentration indices of stunting, underweight, and wasting. The frequency of stunting among Iranian children was more than that of underweight and wasting. Moreover, a similar pattern of the frequency of malnutrition indices was also observed in terms of residential area.

**Table 2:** Frequency and concentration index of under- five child malnutrition, 2010

***Variable***	***Stunting***		***Underweight***		***Wasting***	
	***Frequency (%)***	***Concentration Index***	***Frequency (%)***	***Concentration Index***	***Frequency (%)***	***Concentration Index***
National	10.13	−0.177 ***	5.7	−0.092***	3.29	−0.031
Urban	8.47	−0.176***	4.94	− 0.073***	3.46	−0.031
Rural	12.99	−0.107***	7.01	−0.094***	3	−0.053

Statistical significance were marked as

* =*P*<0.10,

** =*P*<0.05,

*** =*P*<0.01

The obtained concentration indices of stunting, underweight, and wasting were respectively −0.177, −0.092, and −0.031 at the national level. Socioeconomic inequality in stunting and underweight was statistically significant and the children in the lowest quintile were bearing greater burden of malnutrition (*P*-value<0.01); however, this socioeconomic gradient was not observed in wasting (*P-*value>0.1). The concentration index value in terms of the area of residence showed that the urban children in the lowest quintiles were suffering from greater burden of stunting compared to the rural ones. In contrast, the concentration indices for underweight and wasting in rural areas were somewhat greater than urban areas.

### Determinants and decomposition of child malnutrition

The results of the regression analysis and decomposition of socioeconomic inequality in stunting and underweight are provided in this section. Since the concentration index of wasting was not statistically significant, we did not present its decomposition results.

In order to do the decomposition, the effect of independent socio-economic variables on malnutrition was first evaluated through a multivariate regression model. Then, the distribution of explanatory variables was measured by the concentration index. Finally, the contribution of each independent variable in socio-economic inequality of stunting and underweight was calculated.

Tables 3 and 4 respectively show the coefficient values of stunting and underweight regression. Long-term growth retardation and underweight increased with the increase of children’s age and the number of mother’s deliveries. The probability of chronic malnutrition and underweight was higher in families with two or more children under five years of age.

Furthermore, the probability of stunting and underweight among children declined with the increasing levels of maternal education. For example, the risk of malnutrition in children whose mothers had university degrees was lower than children whose mothers had elementary or junior education levels. In addition, the impact of increased maternal age on reduced stunting was significant while no significant relationship was found between gestational age and the risk of being underweight.

**Table 3:** Determinants and decomposition results of height-for-age scores, 2010

***Variable***	***Coefficient***	***Elasticity***	***CI_k_***	***Contribution***	***Contribution %***
Sex of child					
Female	0.026	0.0206	0.0087	0.0002	−0.0964
Child’s age (months)	0.003***	0.1674	0.0051	0.0009	−0.4639
Birth-order of child	0.080***	0.2892	−0.0626	−0.0181	9.7458
Number of under 5 children					
≥ 2	0.088***	0.0348	−0.1501	−0.0052	2.8148
Mother’s education					
Primary or secondary	−0.178**	−0.1369	0.1281	−0.0175	9.4459
Higher	−0.192**	−0.0345	0.5506	−0.0190	10.2355
Mother’s age at birth (years)					
20 – 29	−0.058	−0.0564	−0.0003	0.0000	−0.0101
30 – 39	−0.124**	−0.0609	0.0562	−0.0034	1.8448
≥ 40	−0.208**	−0.0100	−0.0082	0.0001	−0.0440
Area of residence					
Rural	0.153***	0.0924	−0.2530	−0.0234	12.5955
Wealth quintile					
Poor	−0.179***	−0.0593	−0.3986	0.0236	−12.7241
Middle	−0.225***	−0.0742	0.0034	−0.0003	0.1373
Rich	−0.256***	−0.0841	0.4042	−0.0340	18.3209
Richest	−0.370***	−0.1203	0.8022	−0.0965	51.9807
Household size	0.007	0.0527	−0.0214	−0.0011	0.6075

Statistical significance were marked as

* =*P*<0.10,

** =*P*<0.05,

*** =*P*<0.01

**Table 4:** Determinants and decomposition results of weight-for-age scores, 2010

***Variable***	***Coefficient***	***Elasticity***	***CI_k_***	***Contribution***	***Contribution %***
Sex of child					
Female	0.065***	0.0840	0.0087	0.0007	−0.3270
Child’s age (months)	0.005***	0.3984	0.0051	0.0020	−0.9196
Birth-order of child	0.042***	0.2467	−0.0626	−0.0154	6.9276
Number of under 5 children					
≥ 2	0.061**	0.0390	−0.1501	−0.0058	2.6257
Mother’s education					
Primary or secondary	−0.121***	−0.1491	0.1281	−0.0191	8.5743
Higher	−0.188***	−0.0539	0.5506	−0.0297	13.3300
Mother’s age at birth (years)					
20 – 29	−0.024	−0.0775	−0.3986	0.0309	−0.0057
30 – 39	−0.063	−0.1063	0.0034	−0.0004	1.2557
≥ 40	−0.072	−0.1014	0.4042	−0.0410	−0.0205
Area of residence					
Rural	0.065***	0.0624	−0.2530	−0.0158	7.0829
Wealth quintile					
Poor	−0.146***	−0.0775	−0.3986	0.0309	−13.8669
Middle	−0.201***	−0.1063	0.0034	−0.0004	0.1640
Rich	−0.192***	−0.1014	0.4042	−0.0410	18.3927
Richest	−0.313***	−0.1630	0.8022	−0.1307	58.6831
Household size	0.010	0.1196	−0.0214	−0.0026	1.1501

On the contrary, the impact of gender on increased risk of being underweight was significant. However, no significant relationship was observed between sex of child and stunting. Living in rural areas and being in lower socio-economic quintiles also had a significant effect on increased malnutrition.

Tables 3 and 4 likewise show the results from decomposition analysis that included the concentration index, elasticity, absolute contribution, and percentage contribution of each of the determinants against socio-economic inequality in malnutrition. In general, socio-economic characteristics which were more common among the lower quintiles (their concentration indices were more negative) caused a greater increase in malnutrition inequality. Besides, determinants that had greater elasticity were positively associated with socio-economic inequalities in malnutrition.

Nevertheless, a considerable part of socioeconomic inequality among under-five children malnutrition might be explained by socioeconomic status. More than 50% of the inequalities in stunting and about 63% of the inequality in underweight were reflected by socio-economic status.

Another contributor that reflected the inequality in stunting and underweight was the maternal education level (19% in stunting and 22% in underweight). The rest of the inequality in under-five child malnutrition was influenced by the inequality in areas of residence and the number of child deliveries.

## Discussion

This study was conducted beyond the previous studies with the aim of creating a clear picture of the nutritional status of under-five children in socio-economic groups and measuring the inequality in stunting, underweight, and wasting as well as identifying the determinants associated with this inequality through the decomposition of the concentration index using IrMIDHS 2010 data.

Our results indicated that the prevalence of stunting, underweight, and wasting among under-five Iranian children was respectively 10.13%, 5.7% and 3.29%. The concentration indices of stunting and underweight were −0.177 and −0.092, respectively. Finally, decomposition analysis of socioeconomic inequality in under-five children malnutrition showed that the households’ economic status and maternal education were respectively the most important contributors to the socio-economic inequalities in child malnutrition. The findings of this study were consistent with those of the studies carried out in other countries that showed despite the reduction of malnutrition indices, the average socio-economic inequalities among the groups had increased and their concentration indices were negative (4, 8, 19).

Many programs related to malnutrition including the eradication of iodine deficiency disorders through iodized salt or the promotion of breast-feeding have been conducted in Iran through primary health care network after signing Alma Ata Declaration which was considerably successful (24). After that successful experience, the Multidisciplinary National Program for Improving Nutritional Status of Children began with the aim of improving the nutritional status of children under five years of age and reducing malnutrition, especially among economically and socially disadvantaged households (25). After the implementation of the programs, some studies in Iran showed that, over the years, the absolute value of malnutrition indices had decreased in Iran (14, 26). However, our results revealed that nutrition programs in Iran have been partially successful in reducing malnutrition and children in poor households still suffer from malnutrition. The results of decomposition in our study suggested that the households’ economic status was the most important contributor to inequalities in child malnutrition. More than 50% of inequality in stunting and 60% of inequality in underweight were caused by inequalities in socioeconomic status of the families. Our results were in line with the results of other studies that showed household economic status was the most important determinant influencing inequality in malnutrition. The percentage of economic status contribution or its proxy to malnutrition inequality has been reported to be 70% in Mozambique (27), 65% to 70% in Vietnam (9), 46% in Ghana (28), and 50% to 65% in India (3, 7). In comparison with having access to health care, promoting socio-economic status is far more effective on health indices and outcomes, especially on the reduction of child mortality and improving their nutritional status (29–31). Households’ economic status improves the children’s nutrition and their anthropometric outcomes directly by providing foodstuffs and indirectly by affecting other determinants.

Decomposition analysis also revealed that after socio-economic status, maternal education was the second contributor to affect the inequality in stunting and underweight. The risk of growth disorders among children whose mothers had higher education was much lower than those whose mothers had low education levels. According to our study, 16% of inequalities in stunting and 17% of inequalities in underweight were caused by inequality in maternal education levels. Maternal education level was an effective contributor to inequalities in child malnutrition. The contribution of maternal education in stunting was 13% (6). Moreover, 23% of the inequalities in chronic malnutrition and 30% of the inequalities in underweight was due to the inequalities in maternal education level (32). Parents’ education, especially maternal education, plays an important role in children’s health. Educated mothers seek health care more and do more suitable activities to improve the health and nutritional status of their children (33).

Living in rural areas is the third determinant that explains the inequality in malnutrition. Studying 47 developing countries, a significant difference found between nutritional status of the children in rural and urban areas (34). Furthermore, having investigated the socio-economic determinants affecting the nutritional status of children, a significant difference found between urban and rural children’s nutritional outcomes. The disparity was due to the gap in levels of the main determinants; children living in urban areas enjoyed more favorable conditions (35). Generally, rural households are less educated, have less access to safe drinking water and improved drainage systems, and have less access to healthcare services compared to urban households.

However, programs such as National Program for Improving Nutritional Status of Children have failed to properly influence the social determinants affecting the nutritional status of children, especially in poor families, and to gain access to justice in nutritional status (27). Hence, it is necessary for policymakers to initially do comprehensive interventions in order to reduce the average level of malnutrition, especially stunting, and take more targeted measures to eliminate the inequalities in malnutrition based on socioeconomic determinants.

### Limitations

Firstly, since these data were derived from a cross-sectional survey, the causal assessment of dependent and independent variables should be done with caution. Secondly, this study only evaluated the effects of those variables whose data were collected and measured in the survey, while socio-economic inequality in malnutrition may be affected by other variables such as exposure to diseases or food intake. Thirdly, the economic status of the households in this study was measured through the wealth index based on the household assets. Therefore, the contribution of the economic status in malnutrition inequality might be different from the situation in which the economic status of the households is measured by their income, expenditures or consumption levels.

## Conclusion

Children in poor families were suffering from malnutrition, especially stunting, and the average reduction of malnutrition indices at the national level had caused it to be hidden among children in poor families. Children in poor families were suffering more from malnutrition not only due to their weaker economic status but also because of their mothers’ lower level of knowledge and awareness. If government and policymakers seek to solve this public health problem among children, they have to take direct and targeted efforts to eliminate the existing inequalities in the socioeconomic determinants associated with malnutrition.

## Ethical considerations

Ethical issues (Including plagiarism, informed consent, misconduct, data fabrication and/or falsification, double publication and/or submission, redundancy, etc.) have been completely observed by the authors.
